# miR-200a contributes to the migration of BMSCs induced by the secretions of *E. faecalis* via FOXJ1/NFκB/MMPs axis

**DOI:** 10.1186/s13287-020-01833-1

**Published:** 2020-07-25

**Authors:** Mingwei Li, Lifan Wei, Wei Zhou, Zhiyan He, Shujun Ran, Jingping Liang

**Affiliations:** 1 Department of Endodontics and Operative Dentistry, Shanghai Ninth People’s Hospital, College of Stomatology, Shanghai Jiao Tong University School of Medicine, Shanghai, China; 2 National Clinical Research Center for Oral Diseases, Shanghai, China; 3 Shanghai Key Laboratory of Stomatology & Shanghai Research Institute of Stomatology, Shanghai, China

**Keywords:** Bone marrow mesenchymal stem cells, Cell migration, Nuclear factor kappa B, *Enterococcus faecalis*, miRNA, Host-pathogen interactions

## Abstract

**Background:**

Upon migrating to the injured sites, bone marrow mesenchymal stem cells (BMSCs) play critical roles in the repair of bone lesion caused by chronic apical periodontitis. Emerging evidences have shown that *Enterococcus faecalis* is always associated with apical periodontitis, especially refractory apical periodontitis. But the mechanism underlying how *Enterococcus faecalis* affects the migration of BMSCs remains unclear.

**Methods:**

The effects of *Enterococcus faecalis* supernatants on the migration of BMSCs were determined by transwell migration assays. miRNA sequencing was performed to detect the significantly differentially expressed miRNAs of BMSCs. Proteomics analysis was used to detect the protein expression alterations of BMSCs. Luciferase report assays were deployed to verify the targets of miRNA. Western blot analysis was performed to examine the expressions of matrix metalloproteinases-3, matrix metalloproteinases-9, Forkhead Box Protein J1 (FOXJ1), and nuclear factor kappa B (NFκB). The activations of NFκB were detected by luciferase assays with NFκB*luc* reporter.

**Results:**

We found that *Enterococcus faecalis* supernatants could promote the migration of BMSCs. The upregulation of miR-200a-3p in this process contributed to BMSC migration through downregulating its target Forkhead Box Protein J1. Moreover, FOXJ1/ NFκB axis was found to regulate matrix metalloproteinases (MMPs) in this process.

**Conclusions:**

These results above suggest that miR-200a contributes to the migration of BMSCs induced by the secretions of *E. faecalis* via FOXJ1/NFκB/MMPs axis.

## Background

Apical periodontitis, which is characterized by the inflammation and destruction of the apical periodontium, is always caused by the host immune response to microbial infection in the root canal system [1]. Those species originated from intestinal flora, such as *E. faecalis*, have been identified as dominant factors causing apical periodontitis [2]. *E. faecalis* is always isolated as a monoculture in retreated root canals [3]. It belongs to facultative aerobic species and is usually found in secondary infection or post-treatment of apical periodontitis, especially in the refractory inflammation [4]. *E. faecalis* is tolerated to antimicrobials and contains the ability of surviving in a nutrient-deficient environment. Thus, persisting infections in root canal or apical periodontium are always associated with *E. faecalis* [5]. There are various virulence factors produced by *E. faecalis*, such as lipoteichoic acid, aggregation substance protein, and surface adhesion [6, 7]. During the development of pulpitis, *E. faecalis* can invade and colonize at dentinal tubules. Studies on the etiology of refractory apical periodontitis have revealed that *E. faecalis* biofilms in the dentinal tubules contribute to the retaining of apical periodontitis [8].

Since apical periodontitis is characterized by inflammation and bone resorption [9], cells associated with this process should be carefully taken into consideration. Belonging to multipotent stem cells and widely presenting in bone marrow, bone marrow mesenchymal stem cells (BMSCs) can differentiate into osteoblasts, chondrocytes, or adipocytes [10]. Meanwhile, BMSCs have shown certain ability of moving from niche to the peripheral circulation, and further to the target tissues [11]. The recruitment of BMSCs is required for the repair of bone lesion, and the migration of BMSCs is usually attracted by the environmental factors at the site of injury [12]. There are various factors gathering at the injury, including infectious factors and those produced by injured tissues.

With bacterial infection, BMSCs contact with bacterial components and recognize them through the receptors on the cell membrane. Studies on human BMSCs have revealed that lipopolysaccharide (LPS), the cell wall component from gram-positive bacteria, can increase their migration [13], while the synthetic lipopeptide could inhibit the migration of mouse BMSCs [14]. The migration of human dental pulp stem cells were also increased with the stimulation of Toll-like receptor 2 (TLR2) ligands [15]. Studies above remind us that it depends on the type of mesenchymal stem cell in which migration effect would be caused by bacterial components. In fact, it is hard to ensure the regeneration of the tissue without the efficient migration of BMSCs into the injured sites. It requires more acknowledgments in this field to induce BMSCs migrating and generating new tissues.

In recent years, it has been widely accepted that miRNAs play important roles in the biological regulation of stem cells. miRNAs are highly conserved endogenous non-coding RNAs with a length of 19 to 25 nucleotides. They usually act as a negative regulator by binding to the 3′UTR sites of their target mRNAs [16] and further modulate the cell signaling transduction [17]. They also take part in various biological processes, including cell apoptosis, metabolism, migration, and differentiation [18]. Specific miRNAs have been taken as biomarkers and therapeutic targets for their roles in pathological processes and human diseases [19]. A growing number of miRNAs have been explored for their roles in the migration of BMSCs as either inhibitors or activators. Abnormal miRNA expression would also lead to an obvious alteration on the osteogenic differentiation of BMSCs [20]. A previous study has shown that miR-335 overexpression would downregulate the proliferation, migration, and differentiation of human BMSCs [21]. By upregulating the expressions of MMP-2 and MMP-9, miR-21 can promote the migration of BMSCs via the PI3K/Akt pathway [22]. The migration of rat BMSCs could be inhibited by miR-375 via Akt signaling [23].

The objective of this article is to detect miRNAs related to the migration of BMSCs which is induced by *E. faecalis*, the main infection in refractory apical periodontitis. In this study, we investigate the variations of miRNA signaling of BMSCs in response to *E. faecalis* supernatants (EfS) and evaluate how the miRNAs participate in cell migration. We examine and validate a series of miRNAs which are differentially expressed with the stimulation of *E. faecalis* supernatants, and further elucidate that adjustments of the miRNA can regulate the migration of BMSCs. We provide an insight into the mechanism that miR-200a-3p is involved in the NFκB signaling and affects the expressions of MMPs in the migration of BMSCs. Together, the outcomes of this study provide a better understanding on the movement of BMSCs with the invasion of *E. faecalis* in apical periodontitis and suggest a novel way to drive BMSCs to the injured sites and complete tissue regeneration.

## Methods

### Cell culture

To obtain BMSCs, femur bones from rats were dissected, isolated, and flushed with PBS. Bone marrow was aspirated and suspended in PBS with 5% FBS (Gibco). After centrifuged at 400*g* for 5 min and washed twice with PBS, isolations were cultured in DMEM supplemented with 10% FBS, 100 μg/ml each of penicillin and streptomycin under the condition of 37 °C and 5% CO_2_. *Enterococcus faecalis* ATCC33186 was cultured in brain heart infusion (BHI) medium and the growth rate was measured by the optical density at 600 nm.

### Proteomics analysis

After treated with mediums containing EfS or BHI for 48 h, cells were collected and analyzed as previously described [24]. In brief, label-free peptide MS1 intensity-based methods were used to identify the levels of proteins in different groups, and LC-MS/MS analysis was performed on a Q Exactive mass spectrometer (Thermo Scientific). Those proteins with level change > 2 and *p* < 0.05 were taken as upregulated in EfS- vs. BHI-BMSCs. While proteins with level change < 0.5 and *p* < 0.05 were taken as downregulated. Blast2Go (https://www.blast2go.com) were used for the functional annotation of proteins. KEGG (http://www.kegg.jp) was employed to conduct pathway enrichment. Fisher’s exact test was used in GO and KEGG analysis.

### MicroRNA-sequencing

Cells were treated with indicated mediums for 24 h and miRNA sequencing was performed as previously described [24]. In brief, total RNA was obtained with TRIzol reagent, and then small RNA sequencing libraries were conducted. The libraries were quantified with an Agilent 2100 Bioanalyzer. The raw lllumina sequence data were prepared and converted to fastq files.

### Transwell migration assays

The two-chamber transwell system with 8 μm pore size was used in these assays. After treated with indicated medium for 24–48 h, cells were seeded into the upper chamber of the inserts with serum-free medium, while the medium with 10% FBS was filled into the lower chamber. After incubation for 12 h, the inserts were fixed by 100% methanol and subsequently stained with 0.1% crystal violet. The migrated cells on the lower side of the inserts were imaged and counted.

### Scratch wound assays

After obtaining a confluent monolayer, cells were incubated in the serum-free medium for 12 h and then physically wounded with a sterile pipette tip. Before adding culture medium with BHI or EfS, detached cells were washed away with PBS. The scratches were recorded with a microscope at the defined positions after 0 and 48 h. The scratch wound closures, which were expressed as a percentage of scratch surface area covered by migrated cells, were analyzed with ImageJ.

### Western blot analysis

Bone marrow mesenchymal stem cells between passage 3 and passage 5 were seeded in plates and reached 70–80% at 37 °C in 5% CO_2_ incubator. Stimulation was applied as the indicated time. And then cells were washed with PBS and lysed in RIPA with protease inhibitors. Proteins were extracted, separated by SDS-PAGE, and transferred to PVDF membranes. Before probing with the secondary antibodies conjugated with HRP for 1 h at room temperature, the membranes were blocked with 5% non-fat milk and rinsed in the indicated primary antibodies at 4 °C overnight. Primary antibodies against MMP3(#14351, CST), MMP13(#69926, CST), NFκB(#8242, CST), p-NFκB(#3033, CST), β-actin (#4970, CST), and Foxj1 (#ab235445, Abcam) were applied in this study according to the manufacturers’ instructions. Signals were captured using the enhanced chemiluminescence kit and ChemiDoc MP System. β-actin was shown as the internal control.

### Real-time PCR analysis

After treated with indicated culture medium, cells were lysed and the total RNA extract kit was used to obtain the RNA according to the manufacture’s protocol. After converted into cDNA, gene expressions were detected on a light480 real-time PCR system.

### Oligonucleotide transfection

Synthetic miRNA mimics, inhibitors, and negative control oligonucleotides were designed and produced by Ribobio. BMSCs were transfected with miRNA using DharmaFECT Transfection Reagents according to manufacturer’s protocols. Briefly, transfections were conducted when cells reached 50–60% confluence. Total RNA and proteins were obtained after 24 and 48 h, respectively.

### Luciferase assays

After transfected with appropriate plasmids for 48 h, cells were lysed for luciferase assays. Dual-luciferase reporter assay system was used for detection according to manufacturer’s protocol and Renilla luciferase activities were taken as an internal control.

### Statistical analysis

All statistical analyses were performed by Graphpad Prism 8.0. Statistical results expressed in the figures are shown as the mean ± standard deviation calculated from at least three independent experiments. The statistical significance of the differences was analyzed by unpaired Student’s *t* test at a significance level of *p* < 0.05.

## Results

### *E. faecalis* supernatants (EfS) in late stationary phase promote the migration of BMSCs

**Fig. 1:**
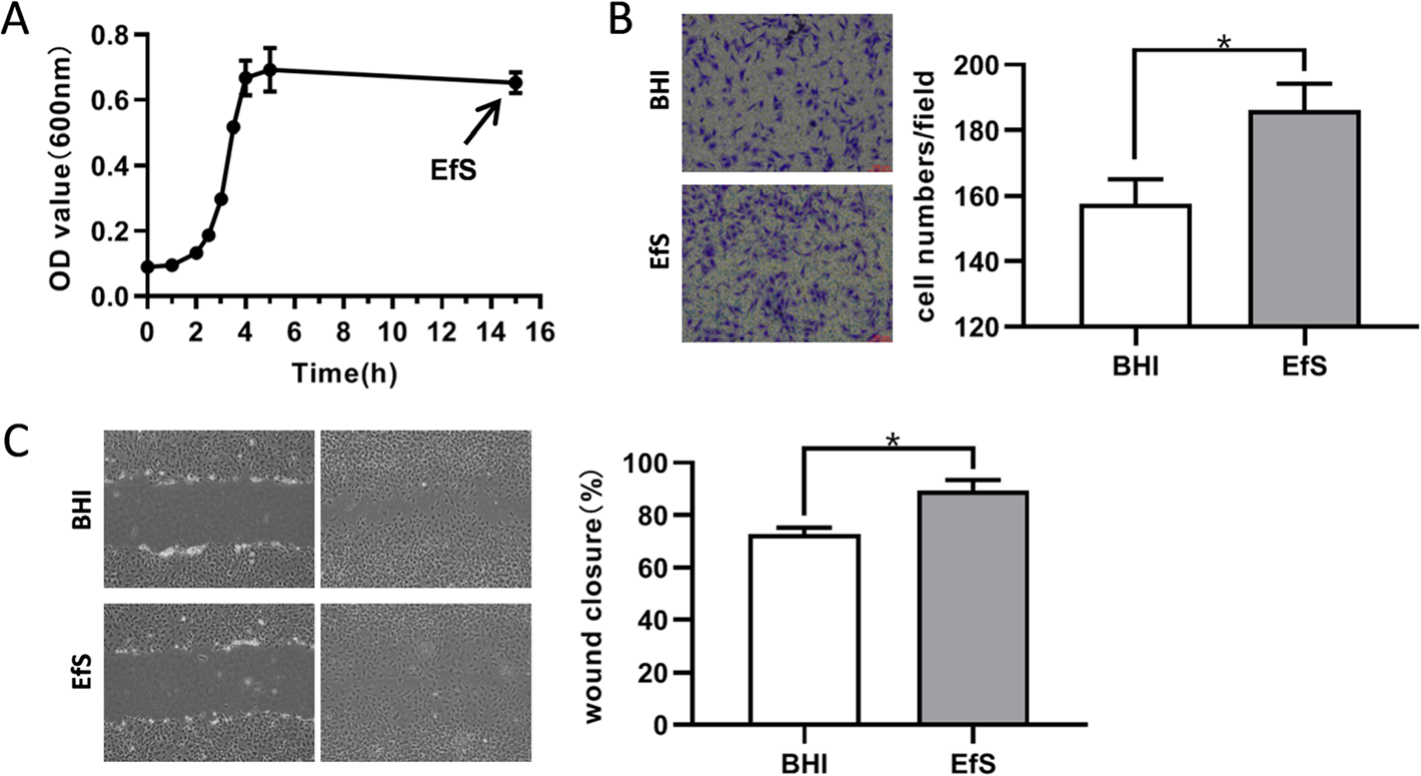
*Enterococcus faecalis* supernatants (EfS) increased the migration of rat bone marrow mesenchymal stem cells (BMSCs). **a** Harvesting of *Enterococcus faecalis* ATCC33186 cell-free culture supernatants. *Enterococcus faecalis* ATCC33186 was cultured in brain heart infusion (BHI) medium and culture supernatants were harvested at late stationary phase. The growth was recorded with the optical density of medium over time. **b** Observation of BMSCs migration with the stimulation of *Enterococcus faecalis* supernatants (EfS) in transwell assays. BMSCs were cultured on the upper inserts of transwell in DMEM with EfS. Those cells migrated to the lower side of transwell membrane were fixed, stained with crystal violet, and counted. **c** Observation of BMSCs migration with the stimulation of EfS in scratch wound assays. The quantitative results are the means ± SD of three independent experiments. *p < 0.05

To address the unsolved questions surrounding how *E. faecalis* affects the role of BMSCs during the repair of periapical bone loss, we obtained the cell-free supernatants of *E. faecalis* ATCC33186, which was cultured in brain heart infusion (BHI) medium. Following cultured in medium for 15 h, the growth of *E. faecalis* was recorded according to the optical density at 600 nm and culture supernatants were harvested in late stationary phase (Fig. 1a). To detect the effect of supernatants on the migration of BMSCs, we performed the transwell assays and scratch wound assays. Compared with the control groups added with BHI medium, the supernatants added into the culture medium dramatically promoted the migration of BMSCs to the lower side of the inserts in transwell (Fig. 1b) and increased the proportion of wound closure area after scratch (Fig. 1c).

### miR-200a-3p participates in the regulation of BMSCs migration

**Fig. 2:**
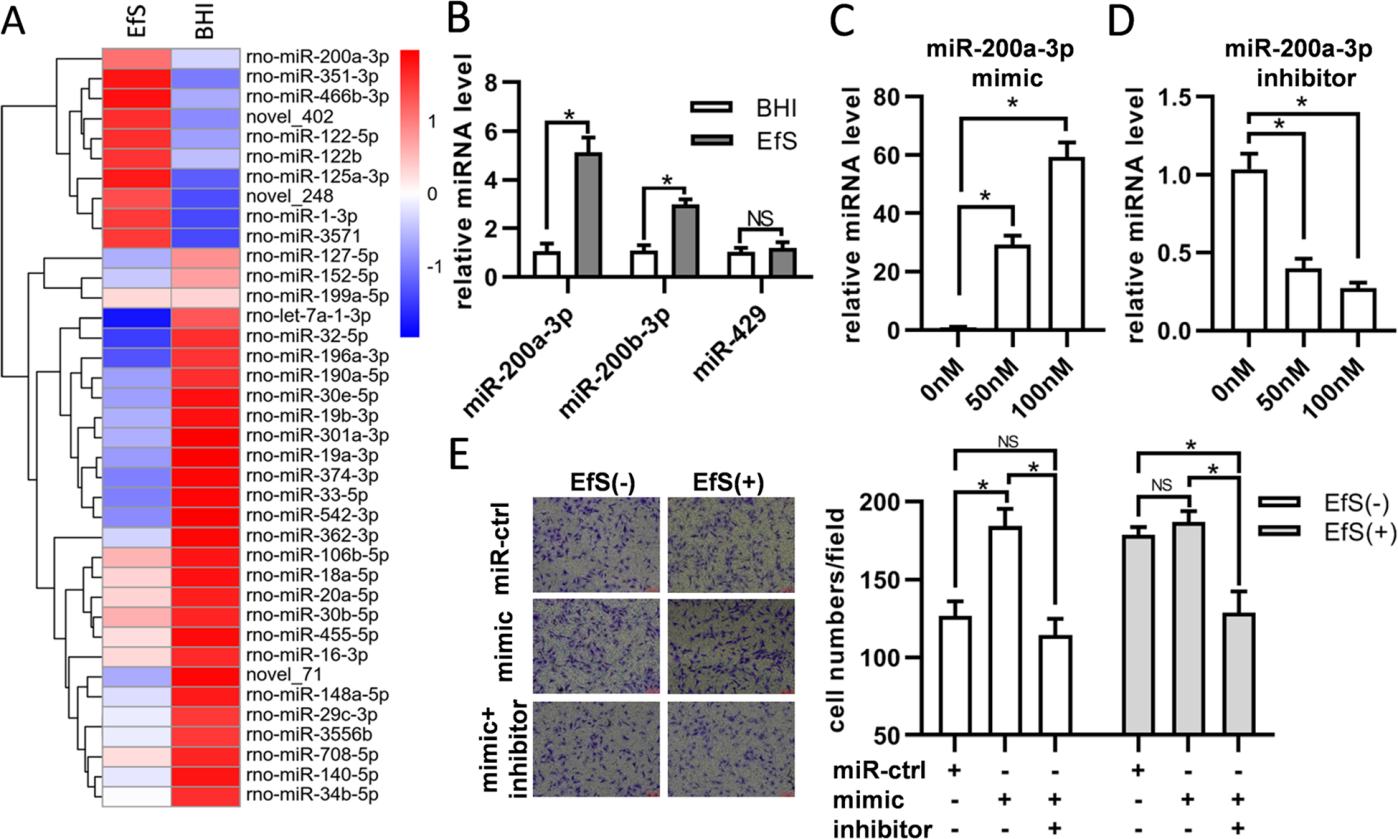
miR-200a-3p was increased in *Enterococcus faecalis* supernatants stimulated BMSCs. **a** Heatmap of differential microRNA (miRNA) expression between BHI and EfS-treated BMSCs. Gene expression data were obtained with miRNA sequence and the most obvious values are shown. Red indicates increase, while blue means decrease. **b** Expression of miR-200 family members determined with qRT-PCR in EfS-treated BMSCs. **c** BMSCs were transfected with miR-200a-3p mimics with the concentration of 50 nM and 100 nM. The expressions of miR-200a-3p were detected by qRT-PCR. **d** BMSCs were transfected with miR-200a-3p inhibitors with the concentration of 50 nM and 100 nM. The expressions of miR-200a-3p were detected by qRT-PCR. In **b**, **c**, and **d**, U6 RNA was taken as internal control. **e** Observation of BMSCs migration with the transfection of miR-200a-3p mimics and inhibitors. After transfection, cells were treated as in Fig. 1b and those cells migrated to the lower side of transwell membrane were counted after 12 h. *p < 0.05; NS, not significant

To further address the question of how *E. faecalis* supernatants impact the migration of BMSCs and whether any miRNAs play roles during this regulation, we performed miRNA sequencing to detect the significantly differentially expressed miRNAs. Thirty-eight miRNAs were defined by setting *p*adj < 0.05 as thresholds and 10 miRNAs were upregulated in BMSCs with *E. faecalis* supernatant treatment (Fig. 2a). Considering that miR-200 family has been proved to take part in the regulation of cell migration, we examined the expressions of miR-200a-3p, miR-200b-3p, and miR-429 by qRT-PCR. The results showed that the expressions of miR-200a-3p and miR-200b-3p were dramatically upregulated, while no significant results were obtained with miR-429 (Fig. 2b). The expressions of miR-200a-3p were also identified after transfection of miR-200a-3p mimics or inhibitors (Fig. 2c, d). To investigate the miR-200a-3p function in BMSCs migration, cells were transfected with miR-200a-3p mimics, inhibitors, or negative control, and transwell assays showed that miR-200a-3p mimics could promote the migration of BMSCs, which could be attenuated by inhibitors (Fig. 2e). With the stimulation of *E. faecalis* supernatants, the migration of BMSCs transfected with miR-200a-3p mimics could still be decreased by miR-200a-3p inhibitors, while no significant result was obtained in comparison with the control group (Fig. 2e).

### Proteomic analysis on the BMSCs treated with *E. faecalis* supernatants

**Fig. 3:**
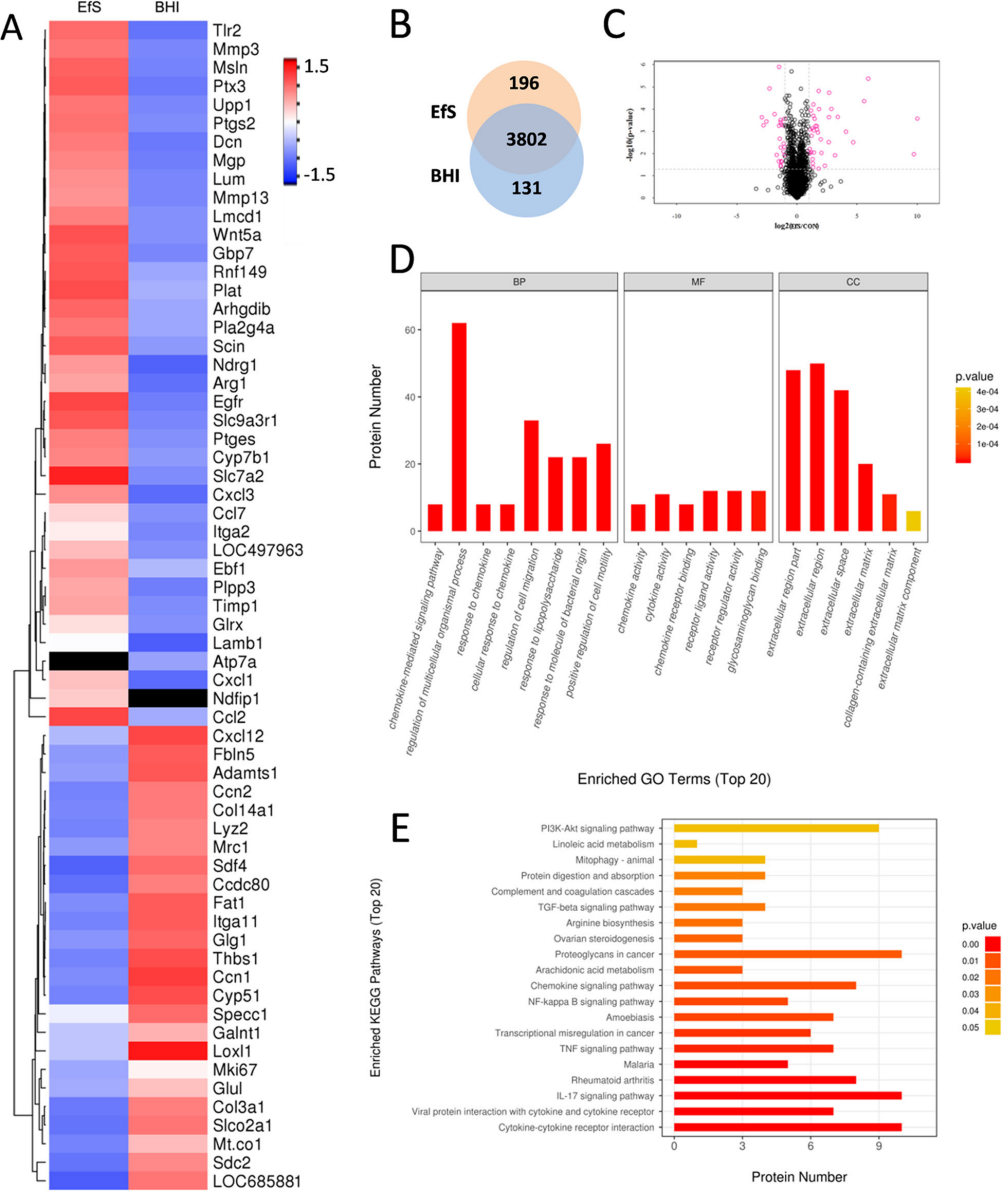
Proteomic analysis on the BMSCs treated with *E. faecalis* supernatants. **a** Differential protein expression heatmap of identified proteins involved in the cell migration. **b** Venn diagram of identified differentially expressed proteins between EfS-treated cells and control. **c** Volcano plot of global differences in protein expression between EfS-treated cells and control (fold change > 2.0; *p* < 0.05). **d** GO enrichment analysis of the differentially expressed proteins related to the cell migration. The top 20 significantly enriched categories are listed. **e** KEGG pathway enrichment analysis of the differentially expressed proteins. The top 20 significantly enriched pathways are listed

Following miRNA detection, we next sought to observe the protein expression alterations of BMSCs during this process. After performing proteomic analysis on BMSCs with or without *E. faecalis* supernatant treatment, we defined 63 significantly differentially expressed proteins by setting absolute level change > 2 and *p* value < 0.05 as thresholds. All the differentially expressed proteins in BMSCs after *E. faecalis* supernatants treatment were listed in the heat map (Fig. 3a). Among these proteins, 38 proteins were upregulated and 25 proteins were downregulated. Next, gene ontology (GO) analysis showed that these proteins participated into a series of biological processes, such as the regulation of cell migration, the response to molecule of bacterial origin, and the positive regulation of cell motility (Fig. 3d). Furthermore, Kyoto Encyclopedia of Genes and Genomes (KEGG) pathway enrichment on the differentially expressed proteins were analyzed, and the top 20 enriched pathways, including NFκB signaling pathway, were shown (Fig. 3e).

### miR-200a-3p affects the expressions of MMP-3 and MMP-13 in BMSCs stimulated with *E. faecalis* supernatants

**Fig. 4:**
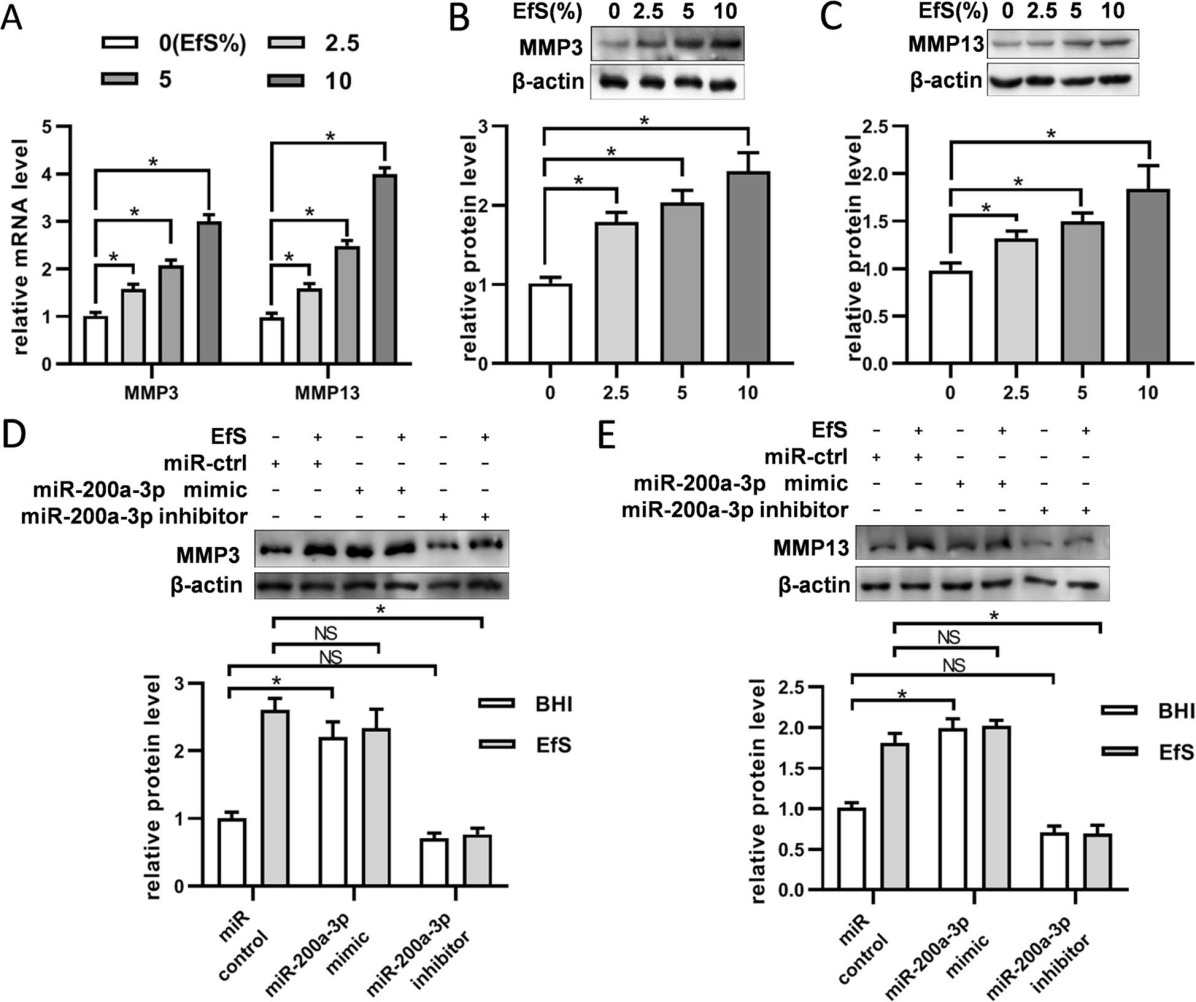
miR-200a-3p regulates MMP-3 and MMP-13 expression in EfS-treated BMSCs. **a** mRNA expressions of MMP-3 and MMP-13 were increased on stimulation of EfS in a dose-dependent manner with 24 h incubation. β-actin was used as internal control. **b** Protein expressions of MMP-3 were upregulated with increasing concentration of EfS after 48 h incubation. **c** Protein expressions of MMP-13 were upregulated with increasing concentration of EfS after 48 h incubation. In **b** and **c**, the levels of β-actin were used as internal control. **d** MMP-3 protein levels were detected by western blot in BMSCs treated with EfS (10%) together with miR-200a-3p mimics (100 nM) or inhibitors (100 nM). **e** MMP-13 protein levels were detected as in **d**. *p < 0.05; NS, not significant

As we chose to focus on the proteins involved in cell migration, further analyses on the mRNA levels of MMP-3 and MMP-13 were performed to identify their alterations in BMSCs with *E. faecalis* supernatant treatment. After cells were stimulated with the indicated concentration of *E. faecalis* supernatants for 24 h, a concentration-dependent manner was obtained at mRNA expression level (Fig. 4a). Compared with the control group, the expressions of MMP-3 and MMP-13 showed a significant upregulation with increasing concentration of *E. faecalis* supernatants. Next, western blot results demonstrated that the expressions of MMP-3 and MMP-13 in BMSCs at protein level could also be promoted by *E. faecalis* supernatants (Fig. 4b and c). Further detection on the protein expressions of MMP-3 and MMP-13 showed that they were obviously upregulated with miR-200a-3p restoration, while inhibition of miR-200a-3p decreased their expressions (Fig. 4d, e).

### miR-200a-3p downregulates the expression of FOXJ1 by binding to its 3′UTR

**Fig. 5:**
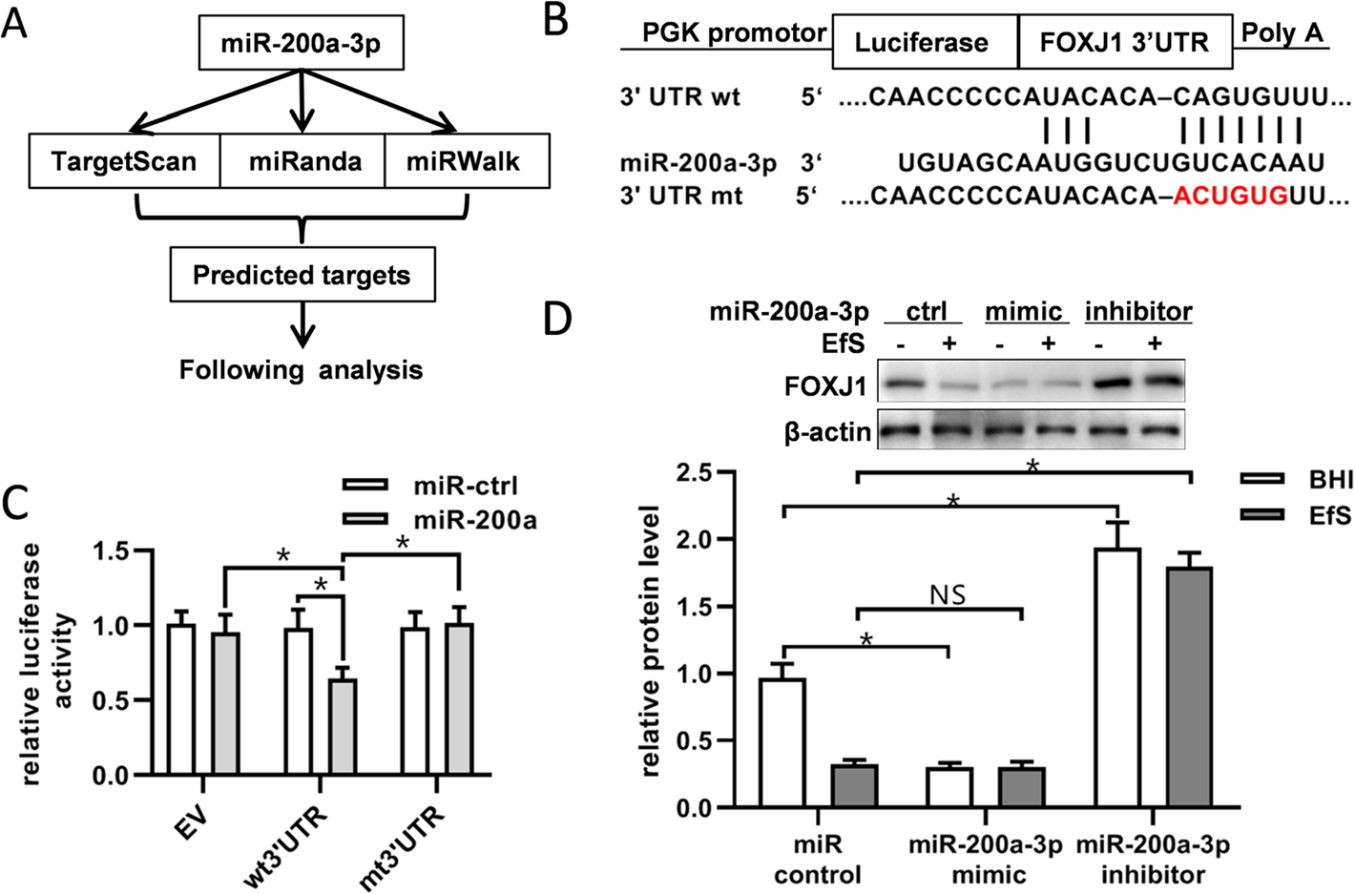
miR-200a-3p decreases the expression of FOXJ1 by binding to its 3′-untranslated region. **a** Prediction on the potential targets of miR-200a-3p using three miRNA target databases. **b** Wild type and mutation of the predicted miR-200a-3p binding sites at the 3’UTR of FOXJ1. **c** The empty vector, wild-type or mutant reporter plasmids were transfected into HEK293T cells, together with miR-ctrl or miR-200a-3p. Luciferase reporter assays were used to identify the binding of miR-200a-3p to FOXJ1 3′UTR. **d** FOXJ1 protein levels were detected by western blot in BMSCs treated with or without EfS (10%), together with miR-200a-3p mimics (100 nM) or inhibitors (100 nM). *p < 0.05; NS, not significant

To further identify the functional target of miR-200a-3p, data were collected through public algorithms, and computational prediction reminded that miR-200a-3p downregulates FOXJ1 expression by directly binding to its 3′UTR (Fig. 5a). To verify the repression of FOXJ1 by miR-200a-3p binding to its 3′UTR, luciferase report assays containing either the wild-type or mutant FOXJ1 3′UTR sequence were conducted (Fig. 5b). The luciferase activities of wild-type FOXJ1 3′UTR reporter were repressed with overexpression of miR-200a-3p, when compared with the control groups (Fig. 5c). Furthermore, we found that BMSCs showed a dramatic decrease of FOXJ1 with *E. faecalis* supernatant treatment (Fig. 5d). No matter with or without *E. faecalis* supernatant treatment, miR-200a-3p mimics could repress the expression of FOXJ1 while inhibitors showed converse function.

### miR-200a-3p increases BMSCs migration through FOXJ1/NFκB pathway

**Fig. 6:**
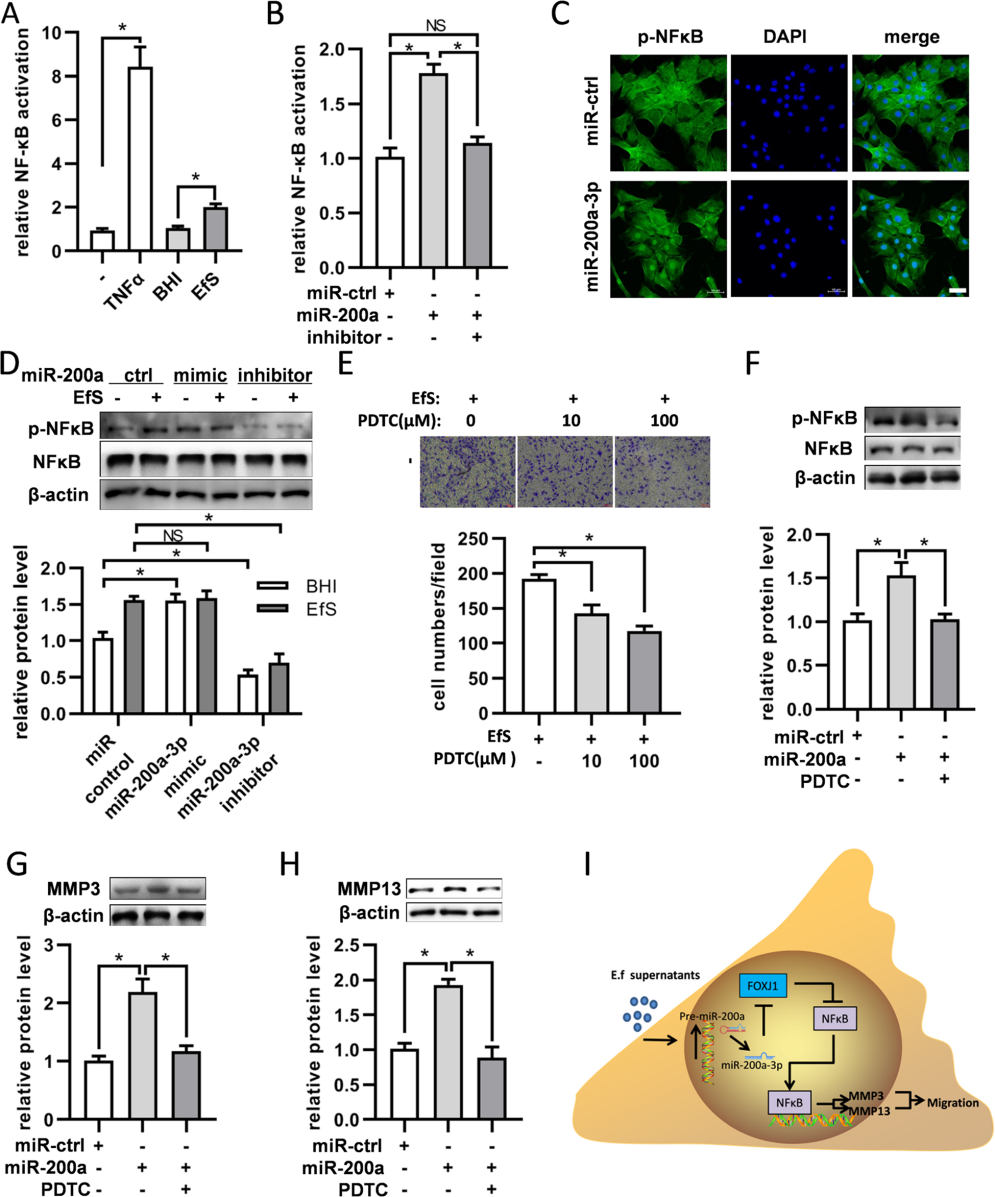
miR-200a-3p affects the expression of MMP-3/MMP-13 through regulating NFκB activity. **a** Increase of the NFκB activity by *E. faecalis* supernatants. The NFκB activation in the BMSCs treated with TNFα, BHI, or EfS were detected by luciferase assays with NFκB*luc* reporter. The TNFα group was used as the positive control. **b** Promotion of the NFκB activity by miR-200a-3p transfection. Luciferase assays were used to detect the NFκB activation of BMSCs after transfection with miR-200a-3p mimics or inhibitors. **c** The expression of p-NFκB was increased in the nucleus of BMSCs with transfection of miR-200a-3p. **d** NFκB and p-NFκB levels were detected by western blot in BMSCs treated with EfS together with miR-200a-3p mimics or inhibitors. Quantification of p-NFκB levels was calculated by normalizing with β-actin. **e** The migration of BMSCs could be attenuated by PDTC under the stimulation of EfS. DMSO was taken as the negative control. **f** Western blot analysis showed the effect of miR-200a-3p on p-NFκB could be repressed by PDTC. Quantifications of p-NFκB level were calculated by normalizing with β-actin. **g** The expression level of MMP-3 increased by miR-200a-3p could be attenuated by PDTC. **h** The expression level of MMP-13 increased by miR-200a-3p could be attenuated by PDTC. In **g** and **h**, quantification was calculated by normalizing with β-actin. **i** Schematic illustration implying the putative signaling pathways involved in the migration of BMSCs induced by *E. faecalis* supernatants. In response to *E. faecalis* supernatants, the transcription of miR-200a-3p is increased. Consequently, the expression of FOXJ1 is attenuated and its inhibition on nuclear factor kappa B (NFκB) pathway is abrogated. Activation and nucleus translocation of NFκB promotes the expressions of MMP-3 and MMP-13, which lead to the increased migration of BMSCs. *p < 0.05; NS, not significant. In **c**, bar = 50 μm

Considering that FOXJ1 acts as a repressor of NFκB activation and the proteomic analysis reminded that the NFκB pathway was included in this process, we sought to investigate whether FOXJ1/NFκB axis plays a role during the migration of BMSCs. Firstly, we determined the level of NFκB activation by luciferase report assays and found that the activation of NFκB was obviously upregulated by *E. faecalis* supernatants (Fig. 6a). Furthermore, transfection of miR-200a-3p could also improve the activity of NFκB (Fig. 6b). Following miR-200a-3p transfection, the expressions of p-NFκB in BMSCs were detected by immunofluorescence. Compared with the control group, cells transfected with miR-200a-3p mimics showed more p-NFκB expression in the cell nucleus (Fig. 6c). Consistent with the expressions of MMP-3 and MMP-13, the expression of p-NFκB could also be increased by miR-200a-3p mimics (Fig. 6d). Meanwhile, transwell assays reminded that inhibition of NFκB with PDTC would attenuate the migration of BMSCs (Fig. 6e). And the upregulation of p-NFκB, MMP-3, and MMP-13 induced by miR-200a-3p mimics could be diminished by PDTC (Fig. 6f–h).

## Discussion

With multiple differentiation potential, BMSCs play an important role during the repair of bone lesion at the injured sites of apical periodontitis. The BMSCs reside in the stem cell niches at bone marrow and it is necessary for them to migrate to the damaged area and differentiate into osteoblasts to regenerate new tissues. Previous studies have described that BMSCs could be chemoattracted by the inflammation factors around the injured tissues [25, 26]. However, it is still unclear whether the migration of BMSCs could be affected by the substances from microbes. Herein, we found that cell-free culture supernatants from the late stationary phase of *E. faecalis* could increase the migration of BMSCs when compared with the BHI medium. It reminds us that the secreted molecules or debris of *E. faecalis* could activate the motility of BMSCs.

A growing number of evidences have shown that miRNAs contribute to the migration of BMSCs under both physiological and pathological situation [22, 27]. To explore whether specific miRNAs were involved in this study, we detected the miRNA alterations of BMSCs treated with *E. faecalis* supernatants by miRNA sequencing. On the top of the list of upregulated miRNAs, miR-200a-3p was noticed. Various studies have demonstrated the participation of miR-200 family in cell migration, and inhibition of miR-200 family members may downregulate the migration of cells [28]. In the present study, the migration of BMSCs was increased after miR-200a-3p mimic transfection, while this promotion could be inhibited when the inhibitors of miR-200a-3p were applied. Together with the results above, these observations provided clues that miR-200a-3p takes part in the regulation of BMSCs migration caused by *E. faecalis*.

Considering that cell motility mainly relies on the protein rearrangements, we performed proteomic analysis to detect the molecular mechanisms underlying BMSCs migration and those proteins with statistical alterations were listed. Among the proteins which were highly expressed in the EfS treated groups, we found MMP-3 and MMP-13 were included. Matrix metalloproteinases (MMPs) belong to a large family and play critical roles in the tissue remodeling and extracellular matrix (ECM) degradation [29]. Various physiological and pathological processes, including cell migration and invasion, require the involvement of MMPs [30]. It has been revealed that MMP-3 and MMP-13 mediate the remodeling of ECM and contribute to the metastasis of cancer cells [31, 32]. Increased expression of MMP-3 and MMP-13 are associated with the augmentation of cell migration in lung cancer [33]. Previous studies have shown that the migration of colorectal cancer cells would be attenuated with downregulating MMP-3 [34], and knockdown either MMP-3 or MMP-13 could repress the invasion and migration of anaplastic thyroid carcinoma cells [35]. At the same time, knockdown of MMP-13 could dramatically downregulate the migration of ESCC (esophageal squamous cell carcinoma) cells [36]. An in vitro study on the adult neural stem/progenitor cells (aNPCs) found an increased expression of MMP-3 during the migration in response to chemokines [37]. Further gene ontology (GO) enrichment analysis on BMSCs also demonstrated that the top 8 affected biological process (BP) contained a response to molecule of bacterial origin, positive regulation of cell motility, and so on. All the results above suggest that molecules in *E. faecalis* supernatants promote the migration of BMSCs through regulating the expression of MMP-3 and MMP-13.

Given that the expression of MMPs showed obvious alteration during the migration of BMSCs induced by *E. faecalis*, we sought to determine the effect of miR-200a on the expression of MMPs. Initially, the expressions of MMP-3 and MMP-13 were confirmed at mRNA and protein level, and their expressions showed a concentration-dependent manner with stimulation of *E. faecalis* supernatants. These results are consisted of what we have obtained in the proteomic analysis. Previous researches have revealed that miR-200 could affect the expression of MMPs [38]. In this study, BMSCs transfected with miR-200a-3p mimics showed similar results with those treated with *E. faecalis* supernatants in the expressions of MMP-3 and MMP-13. When miR-200a-3p inhibitors were applied, there was no obvious difference of MMP-3 and MMP-13 expression in comparison with the control group. However, the inhibitors could diminish the upregulation of MMP-3 and MMP-13 expression induced by mimics or EfS.

miRNA is usually functioned in a unique way, which means that a single miRNA can affect multiple RNA transcripts [39]. Thus, it is necessary to identify the target genes of miR-200a in BMSCs in order to detect the mechanism underlying BMSCs migration. Firstly, bioinformatics analysis was carried out to explore the potential targets of miR-200a-3p. Next, we identified FOXJ1 as one target gene of miR-200a by luciferases reporter assay. Previous studies have demonstrated that miR-200a could increase the migration of non-small cell lung cancer cells [40], while controversial results are also shown that the upregulation of miR-200a could suppress the migration of triple-negative breast cancer cells [41]. All the above reminds us that it may depend on the cell type how the miR-200a affects cell migration. FOXJ1, belonging to a DNA-binding protein family with the forkhead domain [42], could antagonize the NFκB activation by inhibiting IκB protein [43]. Interestingly, we also observed the NFκB signaling pathway in the KEGG analysis.

The NFκB transcription factor family forms various protein complexes and plays critical roles in the control of cell migration [44, 45]. Previous studies have demonstrated that the NFκB/MMP-3 pathway plays roles in various cell migrations, including fibroblasts [46], prostate cancer cells [47], and chondrosarcoma cells [48], and so on. And NFκB/MMP-13 axis contributes to cell migration of lung cancer and glioma [49, 50]. In our present study, both supernatants from *E. faecalis* and miR-200a-3p could promote the NFκB activation, while the inhibitor could suppress the activation. Consistent with the results that the MSC migration induced by IL1β could be impaired by the blockade of NFκB [51], we also found that inhibition of NFκB activation could attenuate the migration of BMSCs, as well as the expressions of MMP-3 and MMP-13. Based on the findings above, we provided evidence that *E. faecalis* supernatants induce the BMSC migration through miR-200a-3p/FOXJ1/NFκB/MMPs axis.

## Conclusions

In this study, we provided evidence on the involvement of miR-200a-3p in the migration of BMSCs induced by *E. faecalis* supernatants and its downstream target FOXJ1. Furthermore, NFκB pathway activation was detected and contributed to the migration by promoting the expressions of MMP-3 and MMP-13 (see Fig. 6i for an overview). These findings provide a new perspective and may help understand the mechanism of BMSC migration in response to *E. faecalis* infection, though further investigations on other miRNAs involved in this process should be concerned.

## Data Availability

All miRNA sequence data supporting the conclusions of this article are available at [NCBI] repository under BioProject No. PRJNA602137. All mass spectrometry proteomics data are available in the [iProX partner] repository, [identifier PXD017438 and the hyperlink to data in http://proteomecentral.proteomexchange.org].

## Abbreviations

aNPCs: Adult neural stem/progenitor cells
BHI: Brain heart infusion
BMSCs: Bone marrow mesenchymal stem cells
DMEM: Dulbecco’s modified Eagle’s medium
EfS: *Enterococcus faecalis* supernatants
FBS: Fetal bovine serum
FOXJ1: Forkhead Box Protein J1
HRP: Horseradish peroxidase
LPS: Lipopolysaccharide
miRNA: Micro ribonucleic acid
MMP: Matrix metalloproteinases
NFκB: Nuclear factor kappa B
PBS: Phosphate buffer saline
PI3K: Phosphatidylinositol 3-kinase
PVDF: Polyvinylidene difluoride
TLR2: Toll-like receptor 2
UTR: Untranslated region
